# Co-localization of plaque macrophages with calcification is associated with a more vulnerable plaque phenotype and a greater calcification burden in coronary target segments as determined by OCT

**DOI:** 10.1371/journal.pone.0205984

**Published:** 2018-10-24

**Authors:** Mathias Burgmaier, Andrea Milzi, Rosalia Dettori, Kathrin Burgmaier, Nikolaus Marx, Sebastian Reith

**Affiliations:** 1 Department of Cardiology, University Hospital of the RWTH Aachen, Aachen, Germany; 2 Department of Pediatrics, University Hospital of Cologne, Cologne, Germany; Brigham and Women’s Hospital, Harvard Medical School, UNITED STATES

## Abstract

**Background:**

The presence of plaque macrophages and microcalcifications are acknowledged features of plaque vulnerability. Experimental data suggest that microcalcifications promote inflammation and macrophages foster microcalcifications. However, co-localization of plaque macrophages and calcification (ColocCaMa) in coronary segments and its impact on plaque phenotype and lesion vulnerability is unexplored.

**Methods:**

Plaque morphology including ColocCaMa of calcified coronary target segments in patients with stable coronary artery disease (n = 116) was analyzed using optical coherence tomography (OCT) prior to coronary intervention. Therefore we considered macrophages co-localized with calcification if their distance in an OCT frame was <100μm and OCT-defined microcalcifications with a calcium arc <22.5°.

**Results:**

ColocCaMa was present in 29/116(25.0%) coronary segments. Calcium burden was greater (calcium volume index:1731±1421°*mm vs. 963±984°*mm, p = 0.002) and calcifications were more superficial (minimal thickness of the fibrous cap overlying the calcification 35±37μm vs. 64±72μm, p = 0.005) in the presence of ColocCaMa. Segments with ColocCaMa demonstrated a higher incidence of newly suggested features of plaque vulnerability, with a 3.5-fold higher number of OCT-defined microcalcifications (0.7±1.0 vs. 0.2±0.6, p = 0.022) and a 6.7-fold higher incidence of plaque inflammation (macrophage volume index:148.7±248.3°*mm vs. 22.2±57.4°*mm, p<0.001). Clinically, intima–media thickness (IMT) in carotid arteries was increased in patients with ColocCaMa (1.02±0.30mm vs. 0.85±0.18, p = 0.021).

In a multivariate model, IMT (OR1.76 for 100μm, 95%CI 1.16–2.65, p = 0.007), HDL-cholesterol (OR0.36 for 10mg/dl, 95%CI 0.16–0.84, p = 0.017), calcium volume index (OR1.07 for 100°*mm, 95%CI 1.00–1.14, p = 0.049), macrophage volume index (OR5.77 for 100°*mm, 95%CI 2.04–16.3, p = 0.001) and minimal luminal area (OR3.41, 95%CI 1.49–7.78, p = 0.004) were independent predictors of ColocCaMa.

**Conclusion:**

Plaque macrophages co-localize with calcifications in coronary target segments and this is associated with high-risk morphological features including microcalcifications and macrophage infiltration as well as with greater calcification burden. Our data may add to the understanding of the relationship between plaque macrophages, vascular calcification and their clinical impact.

## Introduction

Coronary artery disease (CAD) is one of the most relevant pathologies world-wide with a relevant morbidity and mortality, particularly in Western countries [1]. In recent years, research has focused on the progression of coronary atherosclerosis towards acute coronary syndromes (ACS) in search of potential predictors of plaque vulnerability, which may allow clinicians a timely intervention. Among them, features like a lower fibrous cap thickness (FCT) [2,3,4,5], the presence of microchannels [6] or the extension of the necrotic lipid core [2,3,4] have been identified as relevant markers of vulnerable plaques. Besides these established parameters, the presence of macrophages [2,4] and the morphology of calcification [7–13] were recently suggested as novel features of plaque vulnerability. Plaque macrophages reflect plaque inflammation and play a role in lipid accumulation as well as in the disruption of the fibrous components of the plaque inducing a more vulnerable plaque phenotype prone to plaque rupture [14]. On the other hand, both optical coherence tomography (OCT) and intravascular ultrasound (IVUS) have been able to identify small calcifications as features of plaque vulnerability; in particular, Ehara et al. found spotty calcifications, i.e. calcifications with a calcium arc<90° to be more frequently present in culprit lesions of ACS patients rather than in lesions of patients with stable CAD [7]. These findings could be confirmed using the superior resolution of OCT [8,9]. Another interesting aspect of the relationship between calcifications and plaque vulnerability was the hypothesis that the so called microcalcifications may increase the peak circumferential stress of the fibrous cap, thus potentially promoting plaque rupture and triggering ACS [10,11,12]. Interestingly, data from basic science studies suggest that calcifications and macrophages are interconnected: microcalcifications, for instance, promote inflammation *in vitro* and—vice versa—macrophages foster microcalcifications, e.g. via microvesicles [15,16] and induction of an osteogenic phenotype in vascular smooth muscle cells [17].

Despite the known interaction between macrophages and calcifications *in vitro*, there is no clinical data investigating the co-localization between plaque macrophages and calcifications (ColocCaMa) in coronary arteries in living patients. However, the use of OCT enables *in vivo* visualization of both, macrophages and calcifications in coronary arteries. Given the known reciprocal interaction between plaque macrophages and calcifications *in vitro*, this study aimed to quantify the ColocCaMa in the coronary target segments and its implications towards further features of plaque composition and plaque vulnerability using OCT.

## Methods

### Ethics statement

The study was approved by the ethics committee of the University Hospital of the RWTH Aachen (EK 071/11 and EK 277/12) and is in accordance with the declaration of Helsinki on ethical principles for medical research involving human subjects.

### Study population

In this study we enrolled 102 patients with stable CAD, defined as disease without progression of symptoms within the previous 6 weeks, who underwent a planned coronary angiography at the Department of Cardiology of the University Hospital of the RWTH Aachen. A subgroup of this population was object of previously published analyses [13,18]. Prior to intervention and following diagnostic angiography, the 116 target segments were analyzed using OCT. Main criterion of inclusion was the evidence of calcification in the OCT pullback. Exclusion criteria were the localization of the target lesion in the left main coronary artery, in a vessel bifurcation, in a pre–implanted stent or a bypass graft, ACS, pregnancy and acute or chronic kidney disease. Written consent of the patients was obtained.

### OCT image acquisition and analysis

**Fig 1:**
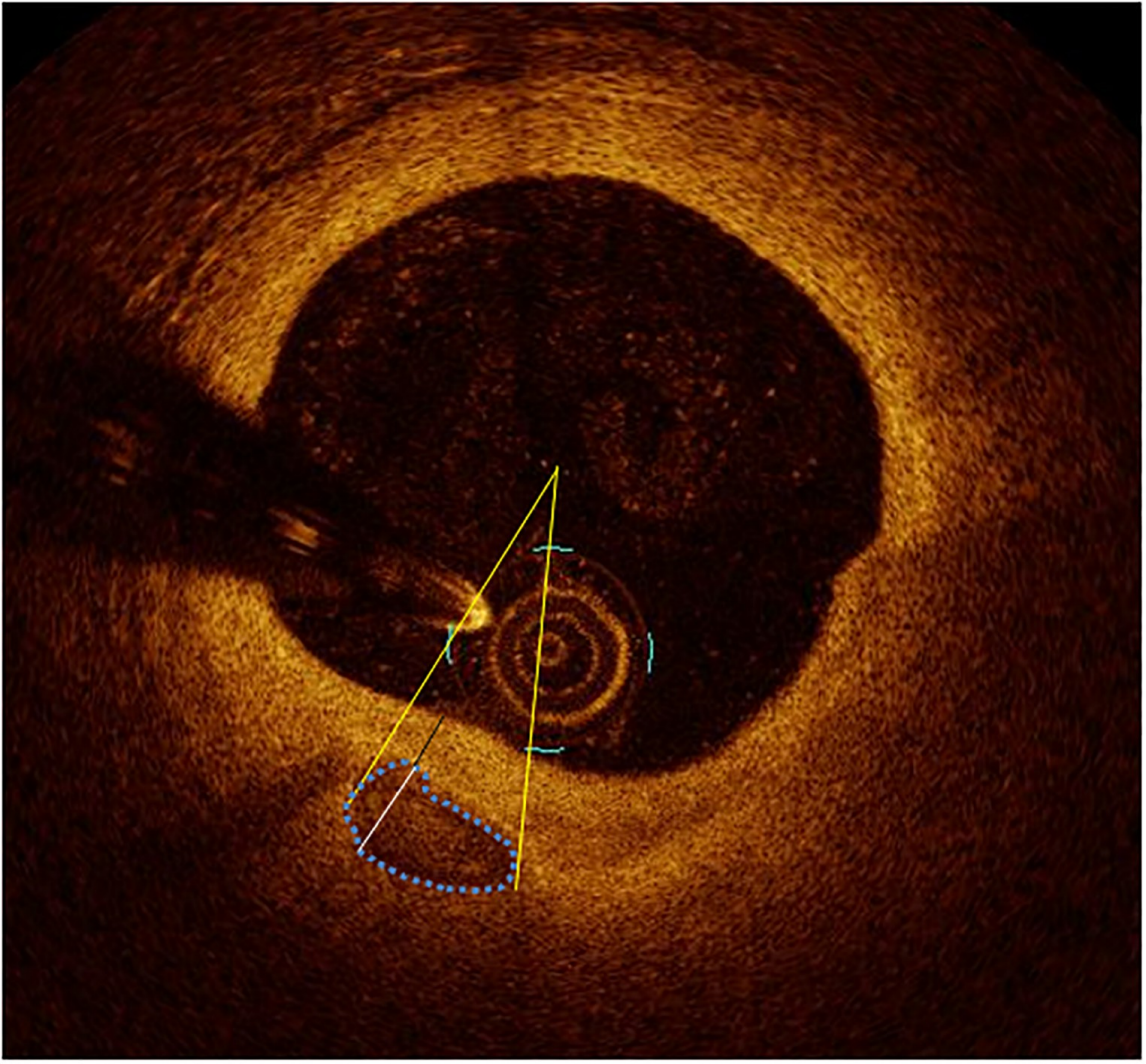
Morphological analysis of calcification using optical coherence tomography. The angle marked by the yellow lines represents the calcium arc. The white line indicates the thickness of calcification, the black one its depth. The blue dotted line shows the calcified area.

**Fig 2:**
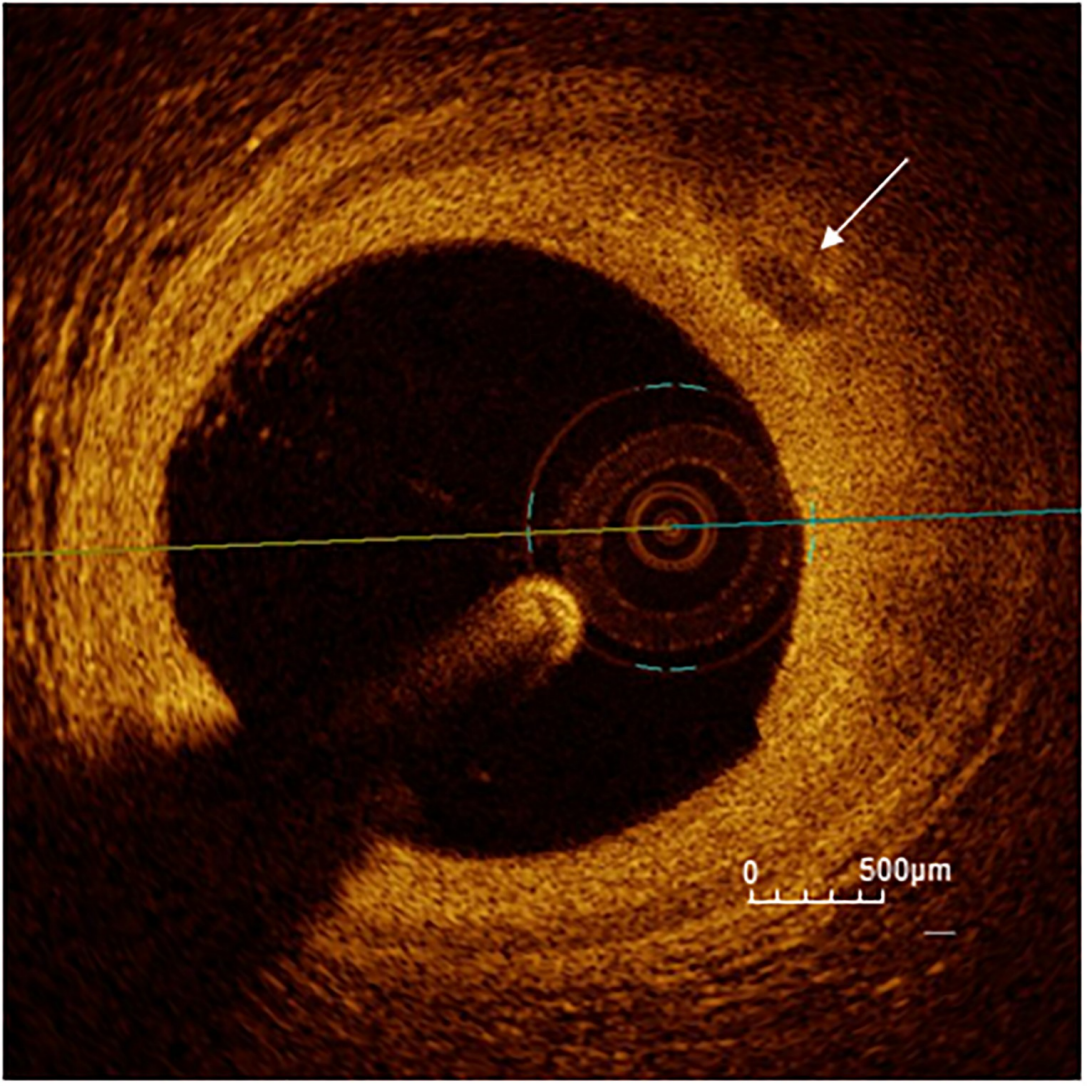
OCT-defined microcalcification. An OCT-defined microcalcification with a calcium arc<22.5° is marked with a white arrow. Scale bar in the right lower corner.

Image acquisition and the analysis of plaque morphology in the coronary target segment were performed as previously described [18]. An example of the analysis is displayed in Fig 1. The analysis was carried out in the whole segment comprising the target lesion, on a frame-by-frame basis in 0.1 mm intervals. In particular, we subdivided calcifications in macrocalcifications with a calcium arc>90°, spotty calcifications with a calcium arc between 22.5° and 90° and OCT-defined microcalcifications with a calcium arc<22.5° as previously published by our study group [18]. An example of an OCT-defined microcalcification is reported in Fig 2. The distance of the more superficial border of a calcification from the lumen was defined as calcium depth. To assess the thickness of calcification and the calcium area in calcifications without a clearly defined deep contour, we used the automatic interpolation function of the commercial analytic software (Ilumien OPTIS Stent Optimization Software, v. E.4, Abbott, Illinois). The smallest calcification in a given target segment was defined as the one having the smallest maximal calcium arc.

**Fig 3:**
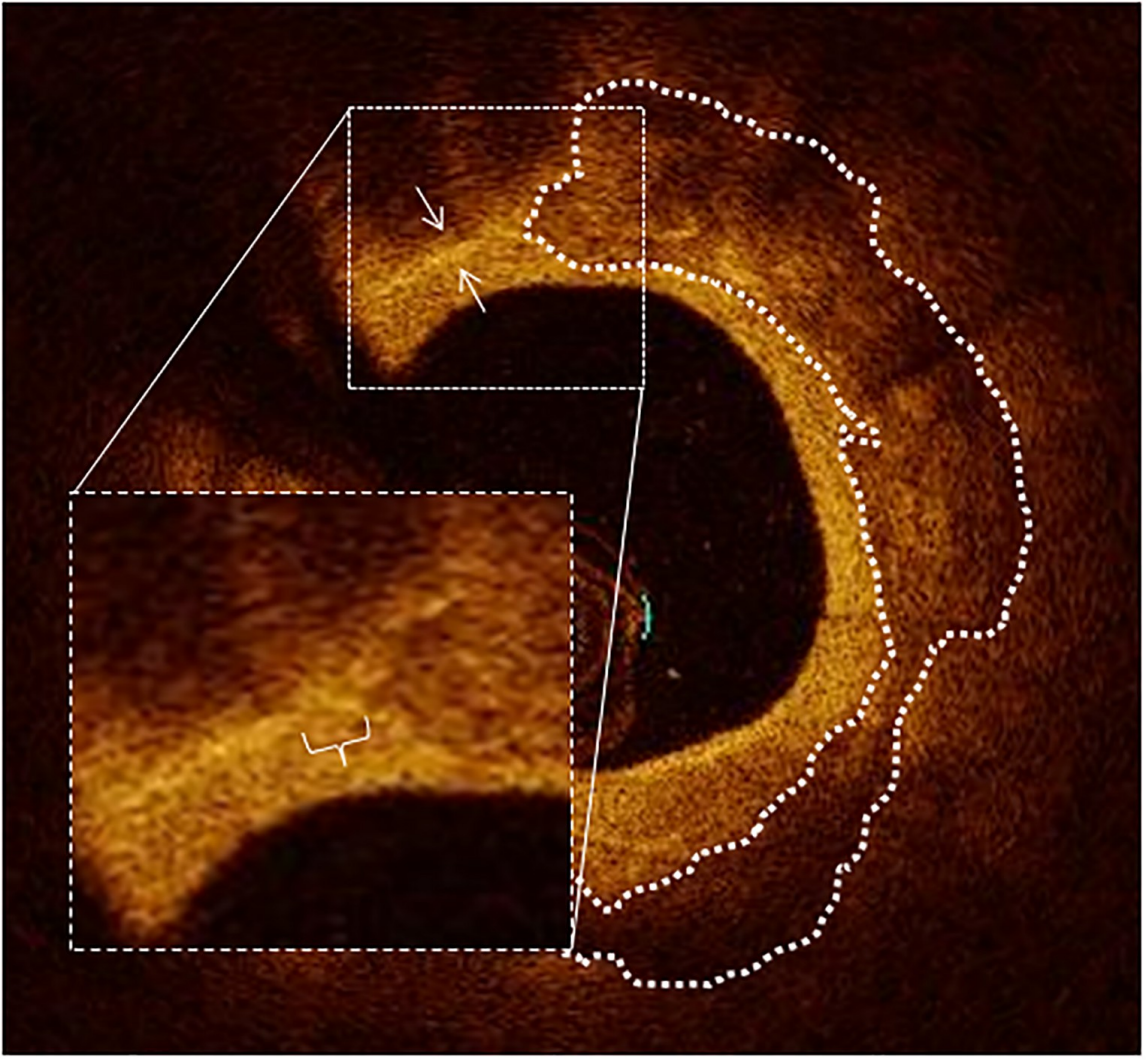
OCT image of a co-localization between macrophages and calcification. Macrophage accumulation is highlighted with two white arrows, macrocalcification is contoured with a white dotted line. In the magnified section, the distance of 90μm (below the defined threshold for co-localization of 100μm) between macrophages and calcification is shown.

According to currently accepted definition, “signal-rich, distinct, or confluent punctate regions that exceed the intensity of background speckle noise” were interpreted as macrophages [19]. Angular extension and length of macrophages were measured, thus obtaining a “macrophage arc” and a “macrophage length”. The product of average macrophage arc and macrophage length was defined as “macrophage volume index” in analogy to the already used parameters for quantification of the lipid core [3,4] and of calcification [18,20,21]. Macrophages were considered to co-localize with calcification when the reciprocal distance in a single OCT frame was smaller than 100μm; an example is displayed in Fig 3. In order to analyze the position of a ColocCaMa, we assessed the localization in the shoulder region of the plaque, defined as the area immediately adjacent to the interface plaque/normal vessel (i.e. the outer 25% of the plaque).

All measurements were performed by two experienced observers. In case of discordance, a consensus measurement was taken. The inter- and intraobserver variability were respectively 0.979 and 0.893 for calcium arc and 0.989 and 0.902 for calcium area [18].

### Carotid ultrasound

Carotid ultrasound was performed using a Vivid I ultrasound system (General Electric, Boston, MA, USA) and a 4- to 13-MHz transducer (8L-RS). The maximal intima media thickness (IMT) of each side was determined using a computerized software. The larger of the two sides was taken for further statistical analysis.

### Statistical analysis

All statistical analyses were performed with SPSS software (IBM Corp., Armonk, NY, USA). Categorical variables were summarized as count (percentage), continuous variables as mean±standard deviation. Distributions of continuous variables were compared with t-test. The association of categorical variables was evaluated by Pearson’s chi-square test. The statistical tests did not account for the presence of multiple lesions in a single patient. To investigate the diagnostic value of morphologic plaque features to predict the presence of a ColocCaMa, univariate logistic regression analysis was performed. The parameters with a p-value below 0.10 were then studied in a multivariate logistic regression analysis with consecutive backward selection for variables with a p-value below 0.10. Among parameters describing the extent of calcification and plaque macrophage infiltration, calcium volume index and macrophage volume index were chosen for the multivariate analysis. Statistical significance was awarded for p<0.05.

## Results

### Clinical parameters

We divided the 116 calcified target segments in two subgroups according to the absence (n = 87) or presence (n = 29) of a ColocCaMa. Among clinical parameters, IMT values were significantly larger (1.02±0.30mm vs. 0.85±0.18mm, p = 0.021) in the presence of ColocCaMa. Further details are reported in Table 1.

**Table 1:** Clinical parameters of patients with and without a co-localization between macrophages and calcification in the coronary target segment. Abbreviations: BP = blood pressure; BMI = body mass index; COPD = chronic obstructive pulmonary disease; CAD = coronary artery disease; PCI = percutaneous coronary intervention; CABG = coronary artery bypass graft; ASS = aspirin; ACEi/ARB = angiotensin converting enzyme inhibitors/angiotensin receptor blockers.

	Co-Localization (n = 29)	No Co-Localization (n = 87)	p
**Sex (male, n,%)**	19 (65.5)	66 (75.9)	0.276
**Age at inclusion (years)**	69.1±6.9	70.2±8.6	0.532
**Systolic BP (mmHg)**	137.7±23.1	137.7±16.9	0.992
**BMI (kg/m**^**2**^**)**	30.1±5.3	29.1±4.3	0.104
**Diabetes mellitus (n,%)**	17 (58.6)	44 (50.6)	0.452
**Hypertension (n,%)**	25 (86.2)	74 (85.1)	0.880
**Hyperlipidaemia (n,%)**	20 (69.0)	48 (55.2)	0.192
**Nicotin abuse at inclusion (n,%)**	6 (20.7)	17 (19.5)	0.893
**Total packyears**	22.9±30.1	24.6±28.3	0.773
**COPD (n,%)**	4 (13.4)	6 (6.9)	0.252
**Known CAD (n,%)**	11 (37.9)	29 (33.3)	0.652
**Previous PCI (n,%)**	8 (27.6)	24 (27.6)	1.000
**Previous CABG (n,%)**	0 (0)	4 (4.6)	0.240
**Intima-media thickness (mm)**	1.02±0.30	0.85±0.18	**0.021**
**Angina at admission (CCS class)**	2.0±1.0	2.1±0.9	0.741
**Dyspnoe at admission (NYHA class)**	1.9±1.1	1.8±0.9	0.672
**Medication**			
**ASS (n,%)**	25 (86.2)	82 (94.3)	0.161
**ACEi/ARB (n,%)**	22 (75.9)	54 (63.5)	0.224
**Betablocker (n,%)**	22 (75.9)	59 (67.8)	0.414
**Statine (n,%)**	17 (58.6)	52 (60.5)	0.861
**Lab values**			
**Total cholesterol at admission (mg/dl)**	184.0±43,4	198.6±42,8	0.121
**LDL cholesterol at admission (mg/dl)**	114.5±37,1	128.4±37,8	0.094
**HDL cholesterol at admission (mg/dl)**	43.3±10,2	48.0±13,2	0.085
**Triglycerides (mg/dl)**	181.7±125.9	151.5±71.0	0.127
**HbA1c (%)**	6.9±1.9	6.3±1.1	0.055

### Plaque morphology

Segments with ColocCaMa presented a significantly smaller lipid core with a lower lipid volume index (2299±1591°*mm vs. 5817±3769°*mm, p<0.001). Furthermore, minimal luminal diameter (MLD: 1.3±0.4mm vs 1.1±0.2mm, p = 0.021) and minimal luminal area (MLA: 2.1±1.2mm^2^ vs. 1.5±0.7mm^2^, p = 0.017) were greater and % area stenosis was smaller (71.0±11.6% vs. 75.6±9.7%, p = 0.039) in the presence of ColocCaMa. With ColocCaMa we detected significantly more extensive plaque macrophage infiltration (macrophage angle: 42.1±27.3° vs. 12.2±20.4°, p<0.001; macrophage length: 2.6±2.3mm vs. 0.5±1.0mm, p<0.001; macrophage volume index, 148.7±248.3°*mm vs. 22.2±57.4°*mm, p<0.001). Please refer to Table 2 for further details.

**Table 2:** Morphological analysis using OCT in target segments with and without co-localization between macrophages and calcification. Abbreviations: RD = reference diameter, MLD = minimal luminal diameter, RA = reference area, MLA = minimal luminal area, FCT = fibrous cap thickness.

	Co-Localization (n = 29)	No Co-Localization (n = 87)	p
**Maximal proximal RD (mm)**	3.1±0.6	3.0±0.5	0.414
**Maximal distal RD (mm)**	2.9±0.7	2.7±0.5	0.099
**MLD (mm)**	1.3±0.4	1.1±0.2	**0.021**
**Proximal RA (mm**^**2**^**)**	6.8±3.0	6.1±2.2	0.205
**Distal RA (mm**^**2**^**)**	5.9±2.9	5.0±1.9	0.069
**MLA (mm**^**2**^**)**	2.1±1.2	1.5±0.7	**0.017**
**Percent area stenosis (%)**	71.0±11.6	75.6±9.7	**0.039**
**Mean lipid arc(°)**	110.0±33.0	139.4±48.0	0.077
**Lipid plaque length(mm)**	14.5±6.2	15.2±8.9	0.734
Lipid volume index(°*mm)	2299±1591	5817±3769	**<0.001**
**Minimal FCT (μm)**	104±28	89±31	0.166
**Mean FCT (μm)**	137±27	132±32	0.628
**Mean macrophage angle (°)**	42.1±27.3	12.2±20.4	**<0.001**
**Macrophage length (mm)**	2.6±2.3	0.5±1.0	**<0.001**
Macrophage volume index(°*mm)	148.7±248.3	22.2±57.4	**<0.001**

In the analysis of calcification characteristics, segments presenting ColocCaMa showed a higher burden of calcification as expressed by higher average angle of calcification (96.8±56.5° vs. 73.0±31.7°, p = 0.038), length of calcification (16.7±9.0mm vs. 11.1±8.7mm, p = 0.004) and calcium volume index (1731±1421°*mm vs. 963±984°*mm, p = 0.002), as well as a lower minimal cap thickness overlying the calcification (35±37μm vs. 64±72μm, p = 0.005). Furthermore, the total number of calcifications in the plaque (5.0±3.0 vs. 3.5±2.5, p = 0.010) and the number of OCT-defined microcalcifications (0.7±1.0 vs. 0.2±0.6, p = 0.022) were higher in plaques with ColocCaMa. Additional data are reported in Table 3.

**Table 3:** Morphological analysis of calcification in coronary segments with and without a co-localization between macrophages and calcification.

	Co-Localization (n = 29)	No Co-Localization (n = 87)	p
**Presence of microcalcifications (n,%)**	11 (37.9)	11 (11.7)	**0.003**
**No. of microcalcifications (n per segment)**	0.7±1.0	0.2±0.6	**0.022**
**Total no. of calcification (n per segment)**	5.0±3.0	3.5±2.5	**0.010**
**Average calcium arc (°)**	96.8±56.5	73.0±31.7	**0.038**
**Average thickness of calcification (mm)**	0.56±0.16	0.52±0.13	0.213
**Maximal thickness of calcification (mm)**	1.05±0.36	0.93±0.31	0.113
**Minimal depth of calcification (μm)**	35±37	64±72	**0.005**
**Average calcified area (mm**^**2**^**)**	1.2±0.9	0.8±0.6	**0.048**
**Maximum calcified area (mm**^**2**^**)**	3.5±2.3	2.9±6.8	0.675
**Calcium length (mm)**	16.7±9.0	11.1±8.7	**0.004**
Calcium Volume Index (°*mm)	1731±1421	963±984	**0.002**
**Average calcium arc of the smallest calcification (°)**	46.1±66.2	40.1±25.2	0.499
**Average thickness of the smallest calcification (mm)**	0.35±0.21	0.38±0.19	0.449
**Average calcified area of the smallest calcification (mm**^**2**^**)**	0.53±1.03	0.38±0.41	0.559

In 15/29 cases (51.7%) ColocCaMa were localized in the shoulder region of the plaque. Calcifications with ColocCaMa showed a mean calcium arc of 93.2±59.3° and a mean thickness of 0.59±0.22 mm.

### Uni- and multivariate analysis of predictors of ColocCaMa

In univariate logistic regression analysis, IMT, lipid volume index, MLD, MLA, parameters assessing the extent of macrophage infiltration (mean macrophage angle, macrophage length, macrophage volume index) and the extent of coronary calcification (total number of calcifications, number of OCT-defined microcalcifications, average calcium arc, average calcified area, calcium length and calcium index) as well as the minimal depth of calcification were all significant predictors of a ColocCaMa (Table 4).

**Table 4:** Univariate analysis on predictors of a co-localization between macrophages and calcification.

	OR (95% CI)	p
**Intima-media thickness (per 0.1 mm)**	1.45 (1.12–1.87)	**0.005**
**LDL cholesterol at admission (per 10 mg/dl)**	0.90 (0.79–1.02)	0.097
**HDL cholesterol at admission (per 10 mg/dl)**	0.71 (0.48–1.05)	0.090
**HbA1c (%)**	1.33 (0.96–1.83)	0.080
**Mean lipid arc (per 10°)**	0.83 (0.68–1.03)	0.088
Lipid volume index(100°*mm)	0.96 (0.92–0.99)	**0.026**
**Minimal luminal diameter (mm)**	7.86 (1.87–33.0)	**0.005**
**Minimal luminal area (mm**^**2**^**)**	1.95 (1.20–3.18)	**0.007**
**Mean macrophage angle (per 10°)**	1.69 (1.35–2.12)	**<0.001**
**Macrophage length (mm)**	2.76 (1.78–4.01)	**<0.001**
Macrophage volume index(100°*mm)	4.05 (1.97–8.35)	**<0.001**
**No. of microcalcifications**	2.07 (1.21–3.54)	**0.008**
**Total no. of calcification**	1.20 (1.03–1.40)	**0.016**
**Average calcium arc (per 10°)**	1.15 (1.03–1.28)	**0.012**
**Minimal depth of calcification (per 10μm)**	0.92 (0.85–0.99)	**0.022**
**Average calcified area (mm**^**2**^**)**	2.06 (1.12–3.79)	**0.020**
**Calcium length (mm)**	1.07 (1.02–1.12)	**0.006**
Calcium Volume Index (per 100°*mm)	1.06 (1.02–1.10)	**0.004**

Since many of these predictors may be reciprocally influenced, we performed multivariate logistic regression analysis. In this analysis, HDL-cholesterol (OR 0.36 for 10 mg/dl, 95%CI 0.16–0.84, p = 0.017), IMT (OR 1.76 for 100μm, 95%CI 1.16–2.65, p = 0.007), calcium volume index (OR 1.07 for 100°*mm, 95%CI 1.00–1.14, p = 0.049), macrophage volume index (OR 5.77 for 100°*mm, 95%CI 2.04–16.31, p = 0.001) and MLA (OR 3.41, 95%CI 1.49–7.78, p = 0.004) were independent predictors of ColocCaMa (Table 5).

**Table 5:** Multivariate analysis on predictors of a co-localization between macrophages and calcification.

	OR (95% CI)	p
**Intima-media thickness (per 0.1 mm)**	1.76 (1.16–2.65)	**0.007**
**HDL cholesterol at admission (per 10 mg/dl)**	0.36 (0.16–0.84)	**0.017**
**Minimal luminal area (mm**^**2**^**)**	3.41 (1.49–7.78)	**0.004**
Macrophage volume index(per 100°*mm)	5.77 (2.04–16.31)	**0.001**
Calcium Index (per 100°*mm)	1.07 (1.00–1.14)	**0.049**

## Discussion

The main findings of this study in patients with stable CAD are:

1. In target segments with ColocCaMa we found more extensive plaque inflammation, larger calcium burden with more superficial plaque calcifications (i.e. with a lower depth of calcification), more OCT-defined microcalcifications and less advanced lesions with larger MLA and smaller necrotic lipid core. Clinically, patients with ColocCaMa presented larger IMT of carotid arteries.
2. Calcium volume index, macrophage volume index, MLA, HDL-cholesterol and IMT of carotid arteries were independent predictors for ColocCaMa.

In order to minimize cardiovascular events due to CAD, the identification of vulnerable lesions is necessary. Intravascular imaging, such as IVUS or OCT, enables the clinician to gain *in vivo* insight into vulnerable plaque features and to use it for patient care. Although it is known that a lower FCT, the presence of microchannels and a larger necrotic lipid core are features of vulnerable plaques [2–6], plaque vulnerability still remains partly unexplored and new potential predictors are continuously proposed and evaluated. Recently, macrophage infiltration and microcalcifications were suggested as novel features of vulnerable plaques.

Macrophages play a relevant role in the genesis of atherosclerosis, being able to induce accumulation of lipids in the plaque; however, they do also promote plaque vulnerability through the catabolic effect on the fibrous components of the plaque [14]. Plaques showing a high rate of macrophages infiltration are therefore active, possibly rupture-prone entities, even though prospective studies supporting the role of macrophages in future coronary events are lacking. The ability of OCT to effectively individuate macrophages has been discussed. In the pioneering work of Tearney and coll., a high degree of positive correlation between macrophages detected by histology and visual OCT-analysis was first shown [22]. Such an approach established itself as consensus [19] and has since then been widely used in all intravascular imaging studies assessing vascular inflammation as a component of plaque vulnerability. In spite of histologic data showing the absence of macrophages in a minority of the OCT-defined bright spots [23], a recent study employing directional coronary atherectomy showed an excellent performance of OCT in detecting macrophage accumulations, with a sensitivity of 85.7% and a specificity of 88.9% [24]. Therefore and in accordance to the current standards, we employed a visual individuation and quantification of macrophages in this study.

On the other hand, small calcifications have been hypothesized as another novel feature of plaque vulnerability. Intravascular imaging studies showed a higher prevalence of calcifications with a calcium arc<90°, the so called *spotty calcifications*, in lesions of ACS patients [7–9]. Other studies suggested the role of even smaller calcifications, i.e. microcalcifications, in the destabilization of the plaque, provided that their presence in the context of a plaque significantly alters its biomechanics with a sharp increase in the circumferential peak stress on the fibrous cap, which predisposes to rupture [10–12]. A universal definition of microcalcifications is lacking; for instance, Maldonado and coll. focused on a diameter <50μm or <60μm [11,25], whereas Cardoso developed a model including particles with a diameter of 10μm [26]. OCT, with its axial resolution of 10–20μm [27], may not be able to detect the smallest of these calcifications, but still remains the best *in vivo* imaging modality allowing the identification of these features—at least until the implementation of the currently only *ex vivo* available Micro-OCT systems with a resolution of 1μm [28]. Recently, an OCT-based definition of microcalcifications was therefore introduced [13].

Macrophage infiltration and calcifications are however not only linked by their independent contribution to plaque vulnerability, but also by a deeper bond which has its roots in the genesis and evolution of the atherosclerotic plaque. It has been demonstrated that macrophages are able to induce an osteogenic phenotype in vascular smooth muscle cells [17] and to promote calcification in the vessel wall e.g. through the release of calcifying matrix vesicles [15], which then trigger the mineralization via an annexin A5- and protein S100A9-dependent pattern [16]. These small calcified areas may then merge into larger, biomechanically relevant microcalcifications. On the other hand, calcifications themselves are able to foster macrophage infiltration [29], generating what Nadra et al. defined as a vicious cycle of inflammation and arterial calcification [30]. The link between plaque inflammation and vascular calcification has already been demonstrated in murine models through micro-CT [31] and in humans through 18-sodium fluoride PET/CT [32].

Due to the seemingly inextricable interdependence of macrophages and calcifications, we chose to analyze *in vivo* their co-localization, its predictors and its clinical effects.

First, we extended the current knowledge by highlighting a higher calcium burden and a more extensive plaque inflammation in coronary plaques with ColocCaMa. This is in line with the above mentioned experimental data that macrophages promote calcifications and calcifications foster inflammation *in vitro* [15,16,17,29]. Moreover, we observed a higher number of OCT-defined microcalcifications and more superficial calcifications in the presence of ColocCaMa, suggesting a more vulnerable plaque phenotype. The impact of ColocCaMa on plaque vulnerability is also suggested by their frequent localization in the plaque shoulder. The more superficial calcifications in case of ColocCaMa may be explained by the catabolic action of collagens exerted by macrophages [14], which according to previous studies may even be the starting point of the calcifying process [33] and therefore explain also the more extensive calcium burden.

The dimensions of calcifications showing ColocCaMa are striking. With an average calcium arc of 93.2±59.3° and an average thickness of 0.59±0.22mm, these calcifications are not microcalcifications. However, plaque vulnerability is not only limited to the morphology of a single calcification, but is influenced by many features of plaque vulnerability [4]; in this specific case, ColocCaMa is associated with more extensive macrophage infiltration, a higher number of microcalcifications and more superficial calcifications suggesting a more vulnerable plaque phenotype.

Furthermore, we demonstrated that in spite of a higher plaque inflammation and calcification, the coronary plaques expressing ColocCaMa present a smaller necrotic lipid core. This is surprising, as volumetric parameters of the necrotic lipid core are well known morphological risk features, but may partly be due to the natural evolution of the coronary plaque, in which the core itself undergoes a process of calcification [34]. This may also be partly due to the study design including only calcified lesions, which inevitably lead to the exclusion of some lipid-rich plaques. However, coronary segments presenting ColocCaMa showed less advanced lesions with lower MLA and lower degree of stenosis. Given that pathology studies demonstrated an association between macrophage infiltration and the initial phase of atherogenesis [35], such active plaques with ColocCaMa may reflect an early yet vulnerable stadium of coronary atherosclerosis, therefore also with a less extensive lipid content. On the other hand, minimal and mean FCT were not significantly different between lesions with and without ColocCaMa.

We identified as clinical predictors of ColocCaMa a lower HDL-cholesterol and a higher IMT of carotid arteries. However, this is not surprising, as HDL is a widely employed marker of cardiovascular risk and on the other hand IMT represents an easily, non-invasively measurable parameter of systemic atherosclerosis. ColocCaMa, which according to our findings may be considered a marker of a dynamic, high-risk coronary plaque, is therefore associated to other clinical high risk features.

Taken together, these aspects allow to hypothesize that ColocCaMa in the coronary plaques arise in high-risk patients in the early phases of the atherosclerotic process, as demonstrated by less advanced stenotic severity, nevertheless yielding a specific biological activity which rapidly promotes further calcification and inflammation.

However, some limitations of the present study may be taken into account. First of all, although we could identify an association between ColocCaMa and a more extensive plaque calcification as well as a more vulnerable plaque phenotype, due to the retrospective nature of our investigation we are unable to prove causality. Although our work is the first study exploring *in vivo* the interesting field of ColocCaMa in patients with CAD, further data in larger patient cohorts are required to confirm our findings. Moreover, as this investigation focused on calcified lesions, we cannot draw any conclusions about the magnitude of macrophage infiltration in non-calcified plaques. Due to the exclusion of patients with chronic kidney failure because of ethical reasons linked to the increased need of contrast medium for the OCT investigation, we cannot draw any conclusion about this subpopulation. Furthermore, due to patient selection, we cannot extend our conclusions to patients with ACS, who due to their clinic may express a particularly vulnerable plaque phenotype—further studies are ongoing in order to analyze this specific group.

## Conclusion

Plaque macrophages co-localize with calcification in calcified coronary target segments and this is associated with more vulnerable plaque phenotype, greater calcification burden and less advanced lesions. Moreover, this is associated with well-known clinical predictors of systemic atherosclerosis. Our data may add to the understanding of the relationship between plaque macrophages, vascular calcification and their clinical impact.

## Abbreviations

ACS: acute coronary syndrome
CAD: coronary artery disease
CoLocCaMa: co-localization of calcification and macrophages
CT: computed tomography
FCT: fibrous cap thickness
IVUS: intravascular ultrasound
MLA: minimal luminal area
MLD: minimal luminal diameter
OCT: optical coherence tomography
